# Aspirin increases chemosensitivity of colorectal cancer cells and inhibits the expression of toll-like receptor 4

**DOI:** 10.1186/s12935-023-02847-4

**Published:** 2023-01-16

**Authors:** Jun Ying, Haiyang Zhou, Zhiguo Wang, Qing You, Junnan Chen, Hao Lu, Jian Zhang

**Affiliations:** 1 Division of Colorectal Surgery, Department of Surgery, Second Affiliated Hospital of Naval Medical University, 415 Fengyang Road, Shanghai, 200003 China; 2 Department of Gastrointestinal Surgery, Second Affiliated Hospital of Naval Medical University, Shanghai, China

**Keywords:** Aspirin, Colorectal cancer, Chemotherapy resistance, Toll-like receptor 4

## Abstract

**Background:**

Chemotherapy resistance is an important bottleneck affecting the efficacy of chemotherapy in colon cancer. Therefore, improving the chemotherapy sensitivity of colorectal cancer cells is of great significance for improving the prognosis of patients with colon cancer.

**Methods:**

CCK-8 assay was employed to examine the cell viability of colorectal cancer cell lines. Realtime-PCR and western blot were used to explore toll-like receptor 4 (TLR4) expression in colorectal cancer cell lines. The functions of TLR4 in the stemness of the colorectal cancer cell lines were analyzed by infecting cells with lentivirus containing TLR4 siRNA.

**Results:**

We found that aspirin could effectively enhance the chemosensitivity of CT26 and HCT116 colorectal cancer cell lines. Aspirin can also inhibit the stemness of colorectal cancer cell including inhibiting the number of clone formation and reducing the volume and number of cell spheres and inducing the down-regulation of stemness-related genes. Besides that, aspirin also lead to down-regulation of TLR4 expression in colorectal cancer cells. The TLR4 positive colorectal cancer cells demonstrated a higher chemotherapy resistance potential than TLR4 negative colorectal cancer cells. In addition, the stemness of TLR4 positive colorectal cancer cells is stronger than TLR4 negative colorectal cancer cells.

**Conclusion:**

The results of our study indicate that aspirin increases chemosensitivity of colorectal cancer cells and inhibits the expression of toll-like receptor 4.

## Background

Colorectal cancer (CRC) is the third most diagnosed cancer, which has become a major cause of death in developed countries [1]. Currently, surgery and chemotherapy are two major therapeutic approaches for CRC. The 5-year survival rate of CRC remains poor because of the resistance to chemotherapy agent. Therefore, in order to study the mechanism of chemoresistance is important to improving the prognosis of CRC patients.

Aspirin, also known as acetylsalicylic acid (acetylsalicylic acid, ASA), synthesized and marketed in 1897 by Bayer, is daily used as decreasing inflammation, pain and fever, and in the prevention of thrombotic cardiovascular diseases. Aspirin has shown promising anti-cancer effects upon breast, lung, stomach, ovarian and colorectal cancer. Several studies indicated a decrease in the occurrence of CRC upon aspirin consumption, and it was also suggested that daily use of aspirin was associated with a significant reduction in colorectal adenomas in patients with previous incidences of CRC [2, 3]. It has been reported that aspirin not only can reduce the occurrence of CRC but also reduces the risk of metastasis in CRC [4, 5]. A recent report on the chemopreventive effects of aspirin showed that the incidence of colorectal cancer in was significantly decreased [6]. However, the potential of aspirin on chemotherapy resistance of CRC cells remains unclear.

It has been reported that the expression of toll-like receptor 4 (TLR4) is expressed on several kinds of tumor cells and contributes to tumorgenesis, metastasis and development [7, 8]. On one hand, TLR4 as a receptor for lipopolysaccharide (LPS), play a key role in inflammation, anti-bacterial cytokines and antiviral [9]. On the other hand, TLR4 plays a key role in connecting inflammation and cancer invasion and progression [10, 11]. Adenocarcinoma patients with higher TLR4 expression in the stromal compartment had a significantly increased risk of disease progression and relapsed significantly earlier than those with lower expression levels [12]. Beside that, it has been reported that TLR4 may act as a cancer stem cells (CSC) marker, prompting tumor invasion and migration, which contributes to the poor prognosis of HCC [13].

In this study, we observe the role of aspirin on the chemotherapy resistance of CRC cells by CCK8 assay. We employ colony formation assay and spheroid formation assay to investigate the effect of aspirin on the stemness of CRC cells. Furthermore, the potential mechanism of aspirin on the CRC cells chemosensitivity will be explored. At last, we verify the relevant mechanism in TCGA database.

## Materials and methods

### Reagents

Aspirin were purchased from Sigma (St. Louis, MO, USA). Chemotherapeutic agents cisplatin and 5-fluorouracil (5-FU) were purchased from Qilu Pharmaceutical Co., Ltd. (JiNan, Shandong, China).

### Cell culture

The colorectal cancer cell line CT26 and human colorectal cancer cell line HCT116 were purchased from the cell bank of the Chinese Academy of Sciences. The two colorectal cancer cell lines were both maintained in RPMI 1640 culture medium (GIBCO, Invitrogen, Carlsbad, CA, USA) supplemented with 10% fetal bovine serum (FBS; GIBCO, Invitrogen), 100 units/ml penicillin and 100 mg/ml streptomycin in a humidified incubator under 95% air and 5% CO_2_ at 37 °C.

### CCK8 assay

The CRC cells were plated in 96-well plate (5 × 10^3^ cells/well). Aspirin and chemotherapy agents were employed to treat CRC cells. At the end of the experiment, CCK8 solution (CCK-8 equal to 1/10 of the volume of the media) was added in each wells for 1 h and then cell viability was detected at 450 nm by microplate reader.

### Spheroid formation assay

For sphere formation assay, 1000 cells were seeded in Ultra Low Attachment 6-well plates and cultured in DMEM/F12 supplemented with B27, N-2, 20 ng/ml EGF and 20 ng/ml bFGF (all from ThermoFisher). The cell spheres were incubated for two weeks and then examined under a light microscope.

### Colony formation assay

CRC cells were seeded in twelve-well plates at a density of 500 cells per well and cultured at 37 °C for 1–2 weeks. At the end of the incubation, colonies were stained with crystal violet solution (containing 0.1% crystal violet, 20% methanol, and 80% phosphate buffered saline) for 30 min. Wells were rinsed with water followed by air drying and the colonies were counted. Each measurement was performed in triplicate.

### Real-time PCR

Total RNA was isolated using TRIZOL (Invitrogen, Carls-bad, CA, USA) and cDNA synthesis was performed using the PrimeScript RT reagent Kit (Takara, Kyoto, Japan) according to the manufacturer’s instructions. The mRNA expression of stemness markers was quantified by real-time quantitative PCR. Quantitative PCR was performed using SYBR Green PCR Kit (Applied BI) according to the manufacturer’s instructions.

### Western blot

The treated cells were washed with PBS and lysed by RIPA and PMSF at a ratio of 100:1 to obtain the total protein for Western blot. Equal amount of proteins was separated by SDS-PAGE and transferred to polyvinylidene fluoride membrane. After transferring, the polyvinylidene fluoride membrane was blocked in 5% fat-free milk/1 × TBS/0.1% Tween-20 for 1 h at room temperature and then incubated with primary antibodies overnight at 4 °C. And then, the membrane was washed with 1 × TBS/0.1% Tween-20 before incubated with the secondary antibody for 1 h at room temperature. Immunoblots were performed by using the BeyoECL Plus substrate system (Beyotime), followed by washing with 1 × TBS/0.1% Tween-20.

### Cell transfection

Small interfering RNA (TLR4 siRNA) was added according to the manufacturer’s instructions. The sequences specific for TLR4: sense 5ʹ-GATCCCGTTGAAACTGCAATCAAGAGTGTTGATATCCGCACTCTTGATTGCAGTTTCAATTTTTTCCAAA-3ʹ; anti-sense: 5ʹ-AGCTTTTGGAAAAAATTGAAACT GCAATCAAGAGTGCGGATATCAACACTCTTGATTGCAGTTTCAACGG-3ʹ. Briefly, 2 × 10^6^ cells were seeded in 75cm^2^ dish. For each transfection, solution A (6 μl of siRNA duplex into 100 μl siRNA transfection medium) and solution B (6 μl of siRNA Transfection Reagent 100 μl siRNA transfection medium) were added and then incubated 30 min at room temperature. After reaching 70% confluence, medium containing lentiviruses and polybrene (8 µg/ml) was added at a multiplicity of infection (MOI) of 10 and mixed with the cells. Polybrene was used to improve infection efficiency. After incubation for 24 h, supernatants in the wells were replaced by DMEM containing FBS and cultured for 24 and 48 h for subsequent analyses.

### Flow cytometry sorting

The colon cancer cells were staining with PE conjugated TLR4 antibody (Bioss Inc, Massachusetts, USA). TLR4 antibody (20 μl) were added 100 μl of cell suspension (1 × 10^6^ cells/ml) and incubated at 4 °C for 30 min and then the stained cells were analyzed using flow cytometry. Colon cancer cells were sorted according to the expression of TLR4 and then used in subsequent experiments.

### Public database bioinformatics analysis

The correlations between TLR4 and stem cells markers (CD44, ALCAM) expression were evaluated via the GEPIA database (http://gepia2.cancer-pku.cn/#analysis) from the TCGA colorectal cancer data [14]. Scatter plots were used to examine the relationship between TLR4 gene expression levels and CD44, ALCAM expression, as estimated based on RNA-Seq expression profile data. Differences with a p < 0.05 were considered to be statistically significant.

### Statistical analysis

Statistical analysis of the data was done by using GraphPad Prism 4. Each experiments repeat 3 times. Student’s t-test was used to compare mean values between two groups. Final values are expressed as mean ± SEM. A difference of p < 0.05 was considered statistically significant.

## Results

### Aspirin enhanced the chemosensitivity of CT26 and HCT116 colorectal cancer cells

**Fig. 1:**
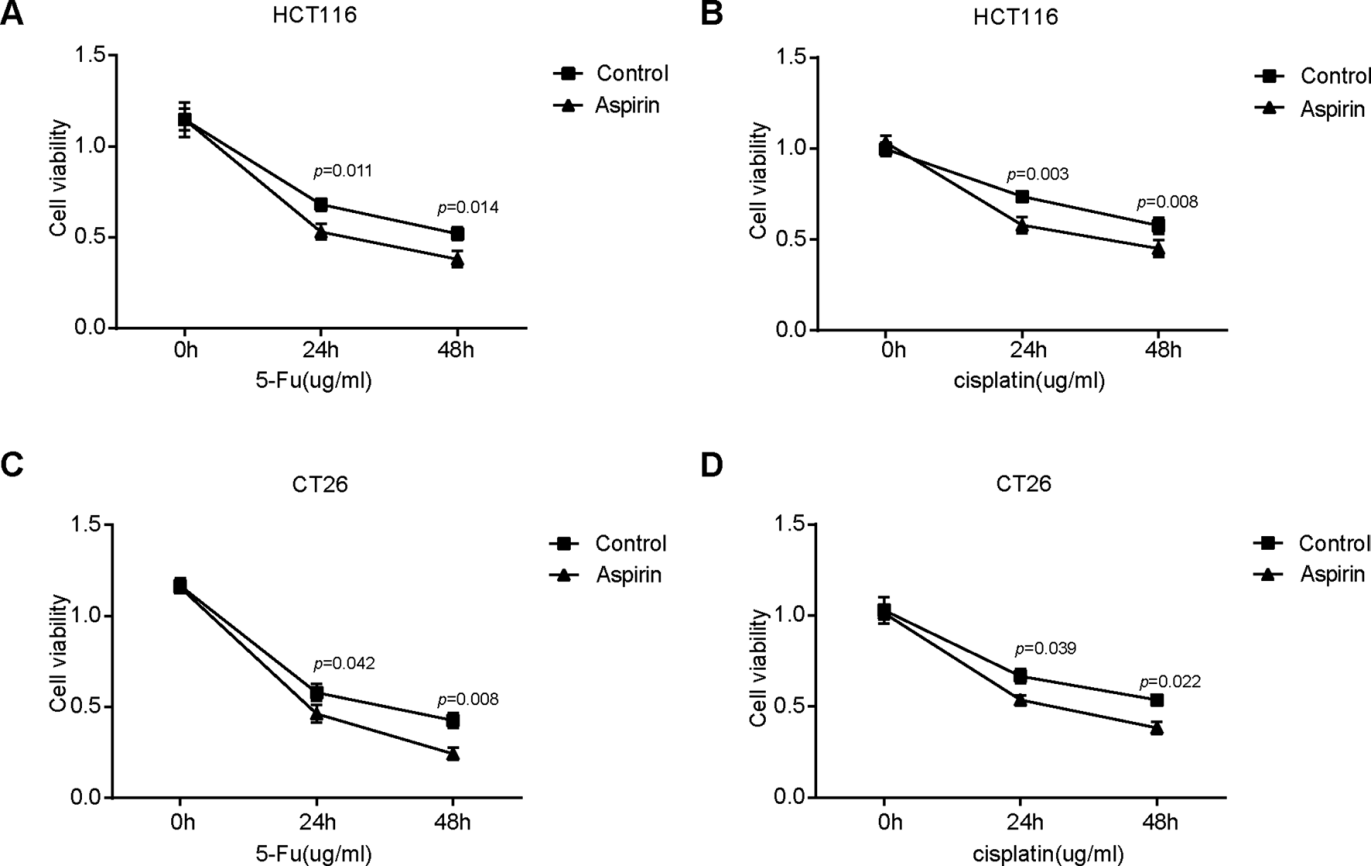
Aspirin enhanced the chemosensitivity of CT26 and HCT116 colorectal cancer cells. **A**–**D** CCK-8 assay was employed the detect the cell viability. CT26 and HCT116 cells were firstly exposed to aspirin (10 mmol/L) for 24 h and then the colorectal cancer cells were treated with 5-Fu or cisplatin for 24 and 48 h. At the end of the experiment, the cell viability in each groups were examined by CCK-8 assay. (*P < 0.05; **P < 0.01)

Firstly, we examined the effect of aspirin on chemosensitivity of CRC cells. CCK-8 assay was employed the detect the cell viability. We found that chemotherapeutic agent chemotherapeutic drugs 5-Fu (10 µg/ml) and cisplatin (5 µg/ml) could effectively decrease the cell viability of CT26 and HCT116 cells. Aspirin treatment alone could not lead to inhibition on the cell viability of CT26 and HCT116 cells. However, compared with control group, aspirin treatment significantly decreased the cell viability of CRC cells with the exposure to 5-Fu and cisplatin (Fig. 1A–D). The results indicate that aspirin treatment could effectively enhance the chemosensitivity of CRC cells.

### Aspirin inhibited the stemness of colorectal cancer cells

**Fig. 2:**
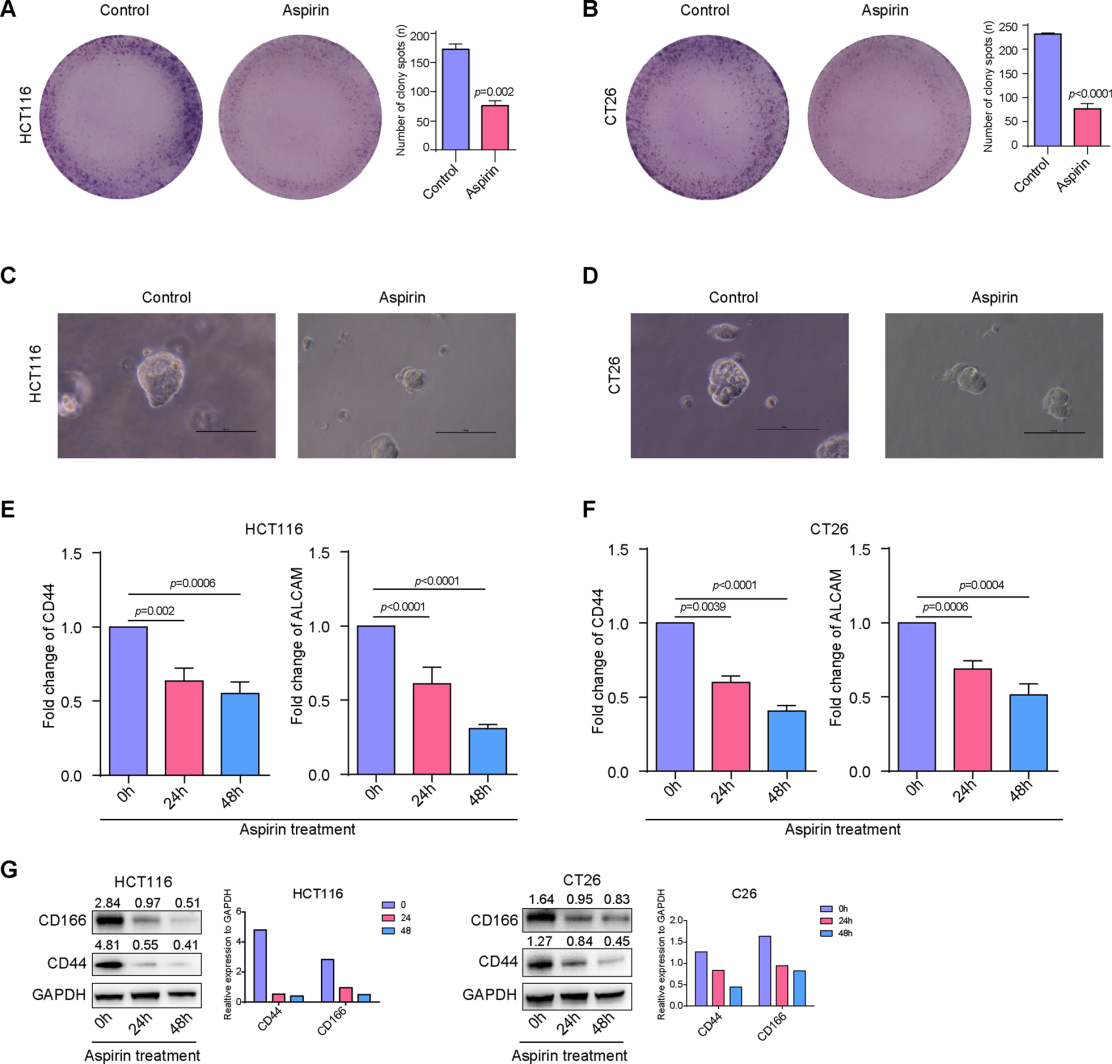
Aspirin inhibited the stemness of colorectal cancer cells. **A**–**B** Cell clone formation assay were used to examine the clone formation potential of CRC cells; **C**–**D** suspension culture was used to examine the cell spheres formation potential of CRC cells; **E**–**F** realtime PCR was employed to determine the expression of stemness-related genes in CRC cells; **G** western blot was employed to determine the expression of stemness-related genes in CRC cells. (**P < 0.01; ***P < 0.001)

The stemness of CRC cells plays an important role in chemotherapy resistance. Then we explored the effect of aspirin on the stemness of CRC cells. Clone formation experiment and suspension culture were used to assess the stemness of CRC cells. As shown in Fig. 2A and B aspirin effectively inhibit the number of clone formation in CRC cells compared with control group. Besides that, the result also demonstrated that aspirin reduced the volume and number of cell spheres (Fig. 2C and D). Furthermore, we examine the expression of stemness associated genes including CD44 and CD166 in CRC cells with or without exposed to aspirin. As shown in Fig. 2E–G, aspirin significantly downregulated the expression of CD44 (24 h, 0.63 ± 0.05, *p* = 0.002, 48 h, 0.55 ± 0.04, *p* = 0.0006) and CD166 (24 h, 0.60 ± 0.02, *p* < 0.0001, 48 h, 0.41 ± 0.02, *p* < 0.0001) at both mRNA and protein level in CRC cells. These results suggest that aspirin can inhibit the stemness of CRC cells.

### Aspirin lead to down-regulation of TLR4 expression of colorectal cancer cells.

**Fig. 3:**
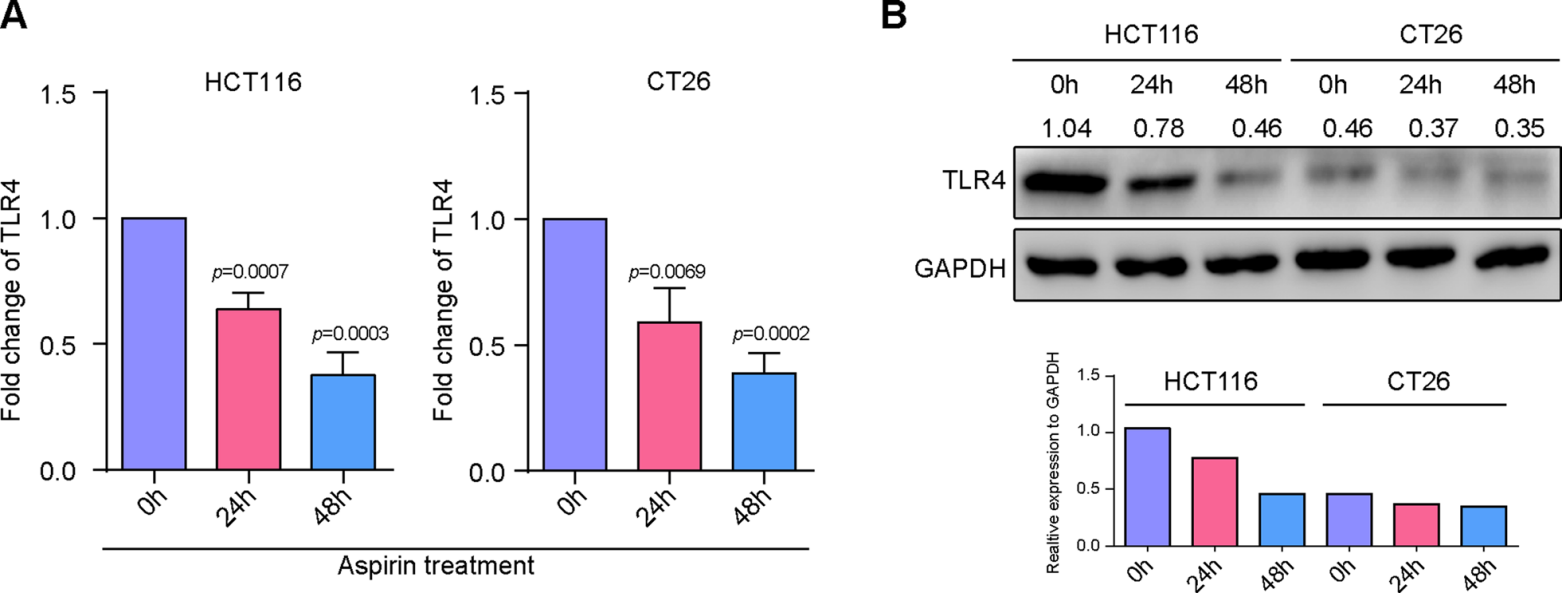
Aspirin lead to down-regulation of TLR4 expression of colorectal cancer cells. **A** realtime PCR was employed to determine the expression of TLR4 in CRC cells; **B** western blot was employed to determine the expression of TLR4 in CRC cells. (**P < 0.01; ***P < 0.001)

Toll like receptor-4 (TLR4) has been reported to play an important role in maintain the stemness of cancer stem cells [15]. Therefore, we observe the role of aspirin on the expression of TLR4 in CRC cells. The results demonstrated that aspirin could down-regulate the expression of TLR4 in CRC cells compared with control group (HCT116: 24 h, 0.64 ± 0.03, *p* = 0.0007, 48 h, 0.37 ± 0.05, *p* = 0.0003. CT26: 24 h, 0.59 ± 0.08, *p* = 0.0069, 48 h, 0.38 ± 0.04, *p* = 0.0002) (Fig. 3A and B), which indicated that aspirin might inhibit the stemness of CRC cells by down-regulating of TLR4 expression.

### TLR4 positive colorectal cancer cells demonstrated a higher chemotherapy resistance potential than TLR4 negative colorectal cancer cells

**Fig. 4:**
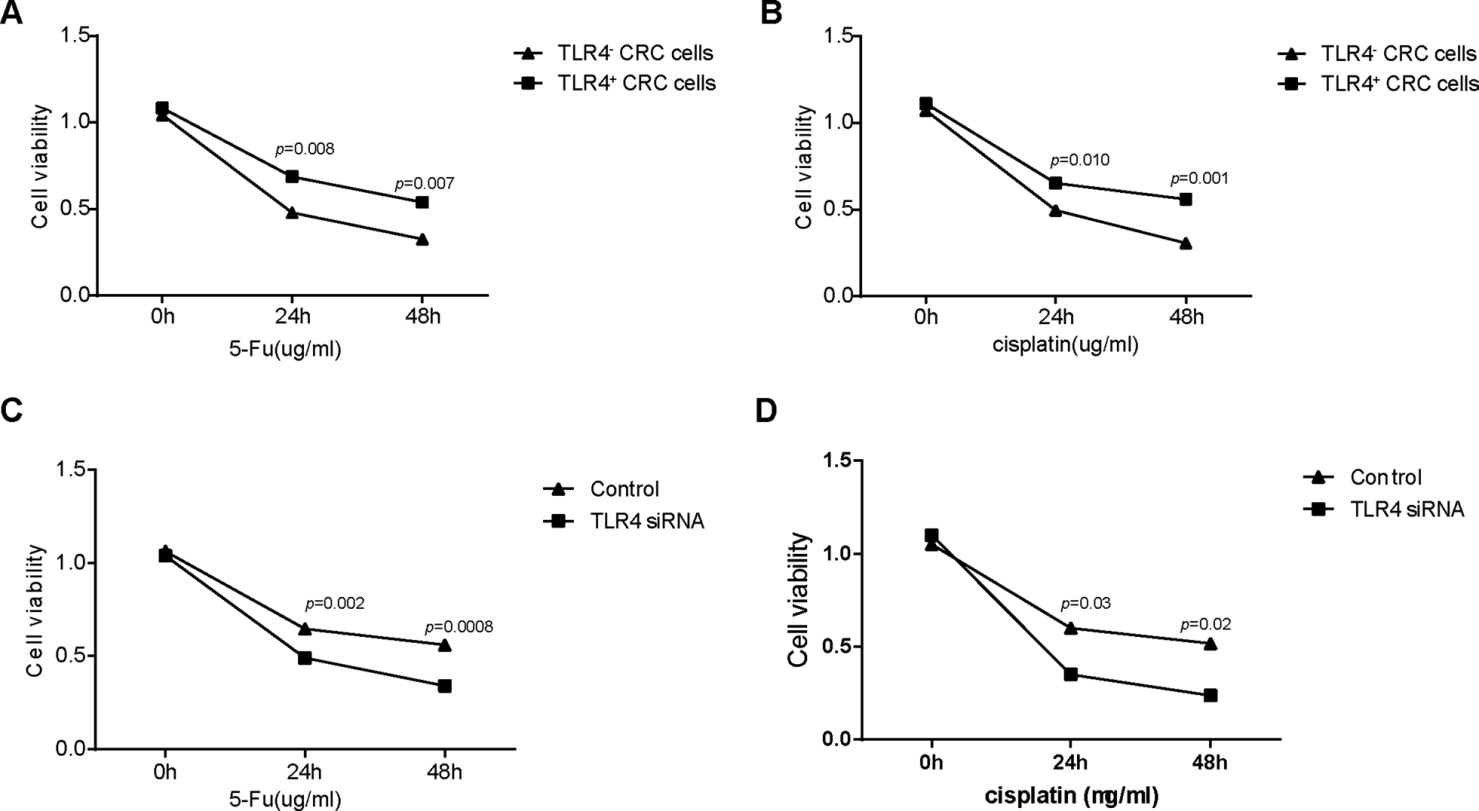
TLR4 positive colorectal cancer cells demonstrated a higher chemotherapy resistance potential than TLR4 negative colorectal cancer cells. **A**–**B** TLR4 positive CRC cells were treated with 5-Fu or cisplatin for 24 and 48 h. At the end of the experiment, the cell viability in each group were examined by CCK-8 assay. TLR4 negative CRC cells were employed as control group; **C**–**D** the expression of TLR4 in TLR4 positive CRC cells were inhibited by siRNA or treated with aspirin and then the cells were exposed by 5-Fu or cisplatin for 24 and 48 h. At the end of the experiment, the cell viability in each group were examined by CCK-8 assay. TLR4 negative and positive CRC cells were employed as control group. (*P < 0.05; **P < 0.01; ***P < 0.001)

To determine the role of TLR4 in aspirin-mediated the chemotherapy resistance of CRC cells, CCK8 assay was employed to examine the cell viability of CRC cells. Firstly, TLR4 negative and positive CRC cells was sorted in HCT116 cells by flow cytometry. The results showed that compared with TLR4 negative CRC cells, TLR4 positive CRC cells demonstrated a higher chemotherapy resistance potential (Fig. 4A and B). We also inhibited the expression of TLR4 in TLR4 positive CRC cells by siRNA and then explored the effect of TLR4 inhibition on chemotherapy resistance. As shown in Fig. 4C, D, inhibition of TLR4 in TLR4 positive CRC cells by siRNA could effectively enhance the chemosensitivity of CRC cells.

### The stemness of TLR4 positive colorectal cancer cells is stronger than TLR4 negative colorectal cancer cells

**Fig. 5:**
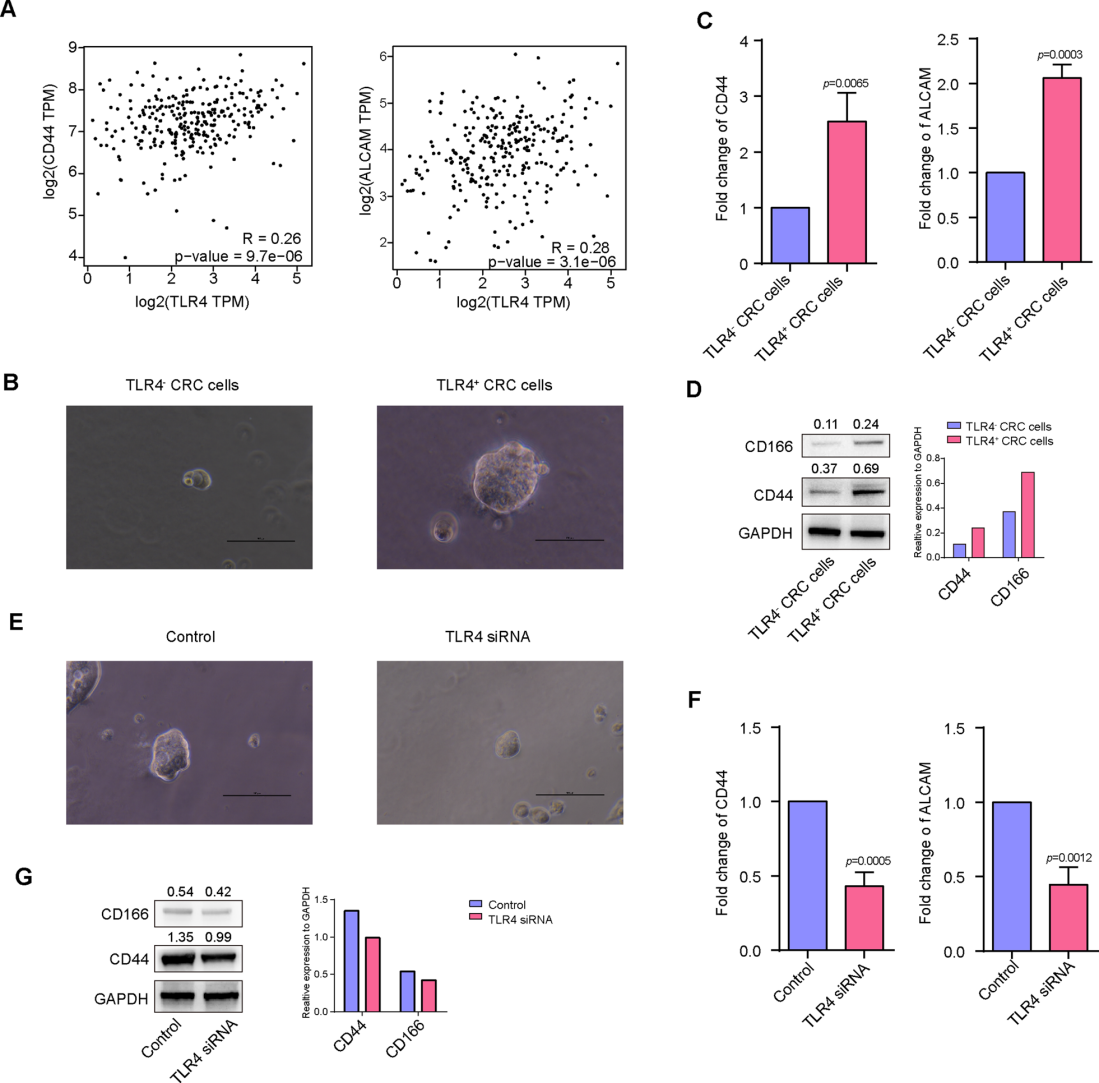
The stemness of TLR4 positive colorectal cancer cells is stronger than TLR4 negative colorectal cancer cells. **A** Correlation analysis between TLR4 and stem cells markers (CD44 and CD166) mRNA level in CRC patients in the TCGA database; **B** cell clone formation assay were used to examine the clone formation potential in TLR4 positive and negative CRC cells; **C** realtime PCR was employed to determine the expression of stemness-related genes in CRC cells; **D** western blot was employed to determine the expression of stemness-related genes in CRC cells; **E** the expression of TLR4 in TLR4 positive CRC cells were inhibited by siRNA or treated with aspirin and then cell spheres formation assay were used to examine the clone formation potential in CRC cells; **F** the expression of TLR4 in TLR4 positive CRC cells were inhibited by siRNA or treated with aspirin and then realtime PCR was employed to determine the expression of stemness-related genes in CRC cells; **G** Western blot was employed to determine the expression of stemness-related genes in CRC cells; (**P < 0.01; ***P < 0.001)

Furthermore, the correlationship between TLR4 and stemness of CRC cells was detected. Positive correlationship between TLR4 and stem cells markers [CD44 (r = 0.26, *p* = 9.7e−06) and CD166 (r = 0.28, *p* = 3.1e−06)] expression were demonstrated in TCGA data of CRC (Fig. 5A). Furthermore, we compared the stemness of TLR4 positive and negative CRC cells. Sphere formation assay was used to assess the stemness of CRC cells. As shown in Fig. 5B, the volume of clones in TLR4 positive CRC cells is higher than TLR4 negative CRC cells. Besides that, we also found that the stemness associated genes expression in TLR4 positive CRC cells is higher than TLR4 negative CRC cells (CD44: 2.54 ± 0.29, *p* = 0.0065. CD166: 2.06 ± 0.08, *p* = 0.0003) (Fig. 5C, D). We then inhibited the expression of TLR4 in TLR4 positive CRC cells by siRNA, the effect of TLR4 inhibition on the stemness of CRC cells was explored. As shown in Fig. 5E, inhibition of TLR4 in TLR4 positive CRC cells effectively lead to decrease of volume of sphere formation. In addition, we found that inhibition of TLR4 in TLR4 positive CRC cells significantly reduced the expression of stemness-related genes (CD44: 0.43 ± 0.05, *p* = 0.0005. CD166: 0.44 ± 0.06, *p* = 0.0012) (Fig. 5F, G).

## Discussion

There are still 40–50% of CRC patients who undergo potentially curative surgery die because of metastatic disease [16]. Therefore, radio and chemotherapy as the main therapeutic approaches to decreasing the recurrence and improving the survival rate of the patients. However, the developed resistance to the chemotherapy in the course of treatment and the inevitable side effects to normal cells seriously limit the application and therapeutic efficacies. Aspirin has been reported that aspirin not only can reduce the occurrence of CRC but also reduces the risk of metastasis in CRC [4, 5]. Besides that, aspirin also could repress the development of osteosarcoma and increase the chemotherapy sensitivity of osteosarcoma by inhibiting NF-κB pathway [17]. In present study, we identified an unexpected function of aspirin in sensitizing colon cancer cells to chemotherapeutic agent 5-Fu and cisplatin treatment. 5-Fu and cisplatin displayed remarkably improved anti-tumor efficiencies in vitro upon co-treatment with aspirin.

Many studies have observed that aspirin can improve the prognosis of digestive malignant tumors. Previous prospective studies have observed that aspirin can reduce colorectal adenomas and reduce the risk of colorectal adenomas recurrence. Aspirin’s preferential effects against CRC compared to cancers of other tissues raises the possibility that it may act directly on colorectal tissues before its absorption into circulation. Aspirin or other NSAIDs have a 40–50% reduced risk of dying from colon cancer, studies demonstrating that treatment of colon cancer cells with aspirin resulted in a concentration-dependent decrease of cell proliferation, migration and invasion [18, 19]. In this study, we examined the effect of aspirin on chemosensitivity of CRC cells, The results indicate that aspirin treatment could effectively enhance the chemosensitivity of CRC cells to 5-Fu and cisplatin.

Cancer stem cells (CSCs) represent a minor subpopulation of the tumor bulk that exhibits self-renewal capacity, induces therapy resistance, and initiates metastasis, thereby promoting tumor progression and recurrence. Therefore, inhibiting the stemness of colon cancer cells may be an important strategy to improve the resistance of colon cancer to chemotherapy. The result of our study demonstrated that aspirin could effectively inhibited the stemness of colorectal cancer cells. LPS plays an important role in occurrence, development and metastasis of tumors [20–22]. In addition, LPS could also increase liver metastasis of human CRC cells [23, 24]. And the function of LPS depends on the expression of TLR4 on the cells [25]. TLR4 as a receptor of LPS has been detected in many human cancer cell lines, including pancreatic, liver and colorectal cancer [20, 23, 26]. And TLR4 plays a key role in connecting inflammation and cancer invasion and progression [10, 11]. Cammarota et al. analyzed 116 tissue samples from patients with different stages of colorectal disease and found that adenocarcinoma patients with higher TLR4 expression in the stromal compartment had a significantly increased risk of disease progression and relapsed significantly earlier than those with lower expression levels [12]. TLR4 was reported to expression in stem cells and cancer stem cells, prompting tumor invasion and migration, which contributes to the poor prognosis of HCC [13, 27–29]. Our previous work have indicated that aspirin inhibited the EMT and the invasive potential of CRC cells by down-regulating LPS/TLR4/NF-κB signaling pathway. In this study, we found that TLR4 positive colorectal cancer cells demonstrated a higher chemotherapy resistance potential than TLR4 negative colorectal cancer cells, which indicated that TLR4 may also serve as a marker of colon cancer stem cells in colon cancer. We further demonstrated aspirin could promoted the chemosensitivity of colorectal cancer cell by suppressing the stemness of CRC cells via downregulating the expression of TLR4.

Furthermore, we employed the TCGA data to analysis the correlationship between TLR4 and stemness of CRC cells. The result demonstrated that relationship between TLR4 and stem cells markers (CD44 and CD166) expression was in CRC tissues, which is consistent with our previous conclusion. These results suggest that TLR4 may be an intervention target to improve the chemosensitivity of colon cancer cells, and on the other hand, clarify the possible mechanism of aspirin to improve the chemosensitivity of colon cancer cells.

Although we have confirmed that aspirin can reduce the stemness of colon cancer cells by inhibiting the expression of TLR4, thus improving the chemosensitivity of colon cancer cells, the potential molecular mechanism by which TLR4 is reduced remains unclear. The scientific question mentioned above has important significance for better aspirin use in the clinical treatment of colon cancer. In future work, we will further study the molecular mechanism of aspirin reducing TLR4 expression, and on this basis, we will observe whether aspirin can have the same effect on other types of tumors.

Taken together, we found that aspirin could effectively enhance the chemosensitivity of CT26 and HCT116 colorectal cancer cell lines. Aspirin can also inhibit the stemness of colorectal cancer cell including inhibiting the number of clone formation and reducing the volume and number of cell spheres and inducing the down-regulation of stemness-related genes. Besides that, we found that aspirin lead to down-regulation of TLR4 expression of colorectal cancer cells. The TLR4 positive colorectal cancer cells demonstrated a higher chemotherapy resistance potential than TLR4 negative colorectal cancer cells. In addition, the stemness of TLR4 positive colorectal cancer cells is stronger than TLR4 negative colorectal cancer cells.

## Conclusions

The results of our study indicate that aspirin up-regulates chemosensitivity of colorectal cancer cells and inhibits the expression of toll-like receptor 4.

## Data Availability

The datasets and materials used and analyzed in this study are available from the corresponding author on reasonable request.

## Abbreviations

CRC: Colorectal cancer
TLR4: Toll-like receptor 4
siRNA: Small interfering RNA
